# Development and evaluation of an end‐to‐end test for head and neck IMRT with a novel multiple‐dosimetric modality phantom

**DOI:** 10.1120/jacmp.v17i2.5705

**Published:** 2016-03-08

**Authors:** Viatcheslav V. Zakjevskii, Cory S. Knill, Joseph. T. Rakowski, Michael G. Snyder

**Affiliations:** ^1^ Wayne State University School of Medicine Detroit MI USA; ^2^ Department of Radiation Oncology Karmanos Cancer Institute Detroit MI USA

**Keywords:** IMRT, radiation therapy, phantom, quality assurance

## Abstract

A comprehensive end‐to‐end test for head and neck IMRT treatments was developed using a custom phantom designed to utilize multiple dosimetry devices. Initial end‐to‐end test and custom H&N phantom were designed to yield maximum information in anatomical regions significant to H&N plans with respect to: (i) geometric accuracy, (ii) dosimetric accuracy, and (iii) treatment reproducibility. The phantom was designed in collaboration with Integrated Medical Technologies. The phantom was imaged on a CT simulator and the CT was reconstructed with 1 mm slice thickness and imported into Varian's Eclipse treatment planning system. OARs and the PTV were contoured with the aid of Smart Segmentation. A clinical template was used to create an eight‐field IMRT plan and dose was calculated with heterogeneity correction on. Plans were delivered with a TrueBeam equipped with a high definition MLC. Preliminary end‐to‐end results were measured using film, ion chambers, and optically stimulated luminescent dosimeters (OSLDs). Ion chamber dose measurements were compared to the treatment planning system. Films were analyzed with FilmQA Pro using composite gamma index. OSLDs were read with a MicroStar reader using a custom calibration curve. Final phantom design incorporated two axial and one coronal film planes with 18 OSLD locations adjacent to those planes as well as four locations for IMRT ionization chambers below inferior film plane. The end‐to‐end test was consistently reproducible, resulting in average gamma pass rate greater than 99% using 3%/3 mm analysis criteria, and average OSLD and ion chamber measurements within 1% of planned dose. After initial calibration of OSLD and film systems, the end‐to‐end test provides next‐day results, allowing for integration in routine clinical QA. Preliminary trials have demonstrated that our end‐to‐end is a reproducible QA tool that enables the ongoing evaluation of dosimetric and geometric accuracy of clinical head and neck treatments.

PACS number(s): 87.55.Qr

## I. INTRODUCTION

Advancements in modern imaging systems allow for very high‐precision, high‐accuracy imaging. Likewise modern external‐beam therapy systems are capable of unprecedented accuracy thanks to improvements in both mechanical precision and dose calculation algorithms. If improved accuracy can be verified in an environment which accounts for heterogeneities typically found in patients, more conformal treatments can be designed with greater dose escalation to the target and improved normal tissue sparing in mind — which could potentially lead to improved patient outcomes.

Current commonly used quality assurance protocols for IMRT and VMAT accuracy make little or no effort to account for anatomical reality. Widely adopted IMRT QA techniques simply recalculate a patient‐specific plan to a geometrically simple homogeneous slab of tissue‐equivalent plastic. These do nothing to verify the treatment planning system's ability to account for heterogeneities, such as bone or air cavities, or realistic patient body contours. Recent publications also suggests that typical IMRT QA techniques have poor performance in detection of treatment delivery errors.^1^, ^2^ IMRT commissioning largely follows similar methodology (i.e., homogeneous phantom, field‐by‐field gamma analysis). The most widely adopted end‐to‐end tests for linear accelerator IMRT are the IROC Houston (formerly RPC) credentialing tests. IROC Houston phantoms (MD Anderson Cancer Center, Houston, TX) are anthropomorphically shaped; however the head and neck phantom does not account for tissue heterogeneities such as bone or air‐filled sinuses. Even with 7% dose‐difference and 4 mm distance‐to‐agreement passing criteria, ~18% of participating institutions fail IROC Houston credentialing.^(3)^

Even in the case where high‐quality commissioning of the planning system and linac has occurred, there is a general lack of standardized QA methods that test the consistency of the entire treatment system, month‐to‐month. QA procedures of the individual components of both the linac and planning system have been well established throughout the field, but these component‐by‐component testing procedures may not elucidate the accumulation of uncertainties throughout the treatment chain, and the complexity of modern treatments seemingly requires a more holistic periodic testing of the overall treatment system. A more thorough, standardized, end‐to‐end IMRT commissioning test that uses clinically realistic structures/plans and an anthropomorphic phantom which accounts for realistic heterogeneities would provide useful, comprehensive information about the state of the treatment chain on a monthly basis.

Realistic end‐to‐end tests have been developed for stereotactic systems. These tests use an anthropomorphic phantom with heterogeneities and a standardized procedure to perform a monthly end‐to‐end test.^4^, ^5^ Currently, conventional linear accelerators lack similar standardized monthly end‐to‐end tests. Up to this point, most in‐house end‐to‐end tests have either been adapted from the stereotactic tests or designed around existing phantoms.^(6)^ These tests typically begin by measuring the accuracy of both static IMRT and RapidArc (Varian Medical Systems, Palo Alto, CA) treatments of simple spherical head tumors ranging from 3 cm to 1 cm in diameter with Gafchromic EBT3 film (Ashland Inc., Covington, KY). A different series of end‐to‐end tests were presented in a recent multi‐institutional study on the commissioning of a number of TrueBeam (Varian) linacs.^(7)^ These tests typically focused on one type of dosimetric modality per phantom and not all phantoms were truly anthropomorphic. Additionally, PTVs in these tests were geometrically simple.

Our goal is to develop new, standardized end‐to‐end testing protocols for conventional linear accelerators, designed to evaluate clinically realistic head and neck plans with multiple dosimetric tools in a novel head and neck phantom which effectively simulates real‐life tissue heterogeneities. Key factors in the design include standardization and reproducibility so that these tests can be successfully incorporated as part of a comprehensive monthly quality assurance program. The standardization of such a test for conventional linear accelerators would allow for the collection of data across multiple centers, and provide a baseline for users to consider when evaluating their own systems in‐house.

## II. MATERIALS AND METHODS

Design of the end‐to‐end test, both in materials and methodology, was guided by the desire to realistically assess the performance of the TrueBeam system in head and neck treatment with regard to geometric and dosimetric accuracy — and to do so with minimal user‐variation in the results. Our test was designed as a true end‐to‐end test which simulates the clinical work flow of simulation, structure contouring, planning, positioning (including image guidance), and delivery of the plan. The creation of a standardized end‐to‐end test for head and neck IMRT involved the creation of a custom anthropomorphic phantom, an anatomical structure set consisting of realistic target and normal tissue volumes, a clinically feasible treatment plan, and a consistently repeatable measurement and assessment methodology.

### A. Phantom design

The initial end‐to‐end test and custom H&N phantom were designed to yield maximum information in anatomical regions significant to H&N plans with respect to: (i) geometric accuracy, (ii) dosimetric accuracy, and (iii) treatment reproducibility. Clinical treatment plans, as well as Radiation Therapy Oncology Group (RTOG) protocols and anatomical atlases, were studied in order to determine regions where geometric and dosimetric accuracy are of greatest importance. Regions of interest included typical PTV locations (oropharynx and neck), as well as critical structures including the spinal cord, parotid glands, and larynx, and the high‐dose gradient regions adjacent to these structures. One major concern was how to place inserts which would not replace heterogeneities with homogeneous soft tissue‐equivalent material, as constructing block inserts to include the same heterogeneity shape/location as the original phantom was not feasible. The final design was a collaborative effort between the authors and Integrated Medical Technologies Inc. (Troy, NY), who manufactured the phantom to the authors’ specifications by heavily modifying a commercially available CIRS phantom (Computerized Imaging Reference Systems Inc., Norfolk, VA). Per the phantom manual, the CIRS phantom is a solid epoxy head phantom, constructed with materials that have linear attenuation coefficients within 1% of anatomical values.

In order to determine geometric accuracy, radiochromic film planes were necessary in likely high‐dose gradient regions; also, orthogonal film planes were desired to measure relative dose distributions in three dimensions. Locations for absolute point‐dose measurements were also seen as vital to ensure dosimetric accuracy. Phantom design would have to meet these design criteria while maintaining true anthropomorphic features, including accurate representation of bony anatomy and air‐filled cavities. Phantom ion chamber and OSLD locations were milled with a vertical machining center (VMC) using dedicated fixtures to localize and hold the phantom pieces. Film planes were created with a crosscut band saw and further refined with the VMC to create a flat surface with holes for dowel pins. Milling with the VMC is a standard machining technique, which has an accuracy of 0.1 mm.

### B. Clinical equipment and software

A Siemens Sensation CT Simulator (Siemens Healthcare, Erlagen, Germany) was used to acquire CT image sets used in the end‐to‐end test. Segmentation and treatment planning were performed with the Eclipse (Varian) treatment planning system. The plan was delivered on a Varian TrueBeam equipped with a high‐definition MLC. Gafchromic EBT3 film was used for planar dosimetry. Film was digitized with an Epson 10000XL scanner (Epson America, Long Beach, CA) and results analyzed with FilmQA Pro (Ashland Advanced Materials, Wayne NJ). Exradin A1SL ionization chamber (Standard Imaging Inc., Middleton, WI) and Landauer nanoDot optically stimulated luminescent dosimeters (OSLDs) were used to determine absolute point doses. OSLDs were read with an InLight microStar reader (Landauer Inc., Glenwood, IL).

### C. End‐to‐end test design methodology

#### C.1 Simulation

The simulation phase of the end‐to‐end test was designed to closely follow established clinical protocols. External crosshair markings on phantom were aligned with lasers and BBs placed on external markings to set isocenter in treatment planning system. CT scan parameters were selected according to established clinical practice for a head and neck scan, with 120 kVp, 320 mAs, and a slice thickness of 1 mm.

#### C.2 Segmentation

In order to ensure reproducibility and standardization of the end‐to‐end test, a standard structure set or standard, reproducible contouring technique was necessary to reduce inter‐user variability in creating structure sets. Smart Segmentation, an automatic protocol included in Eclipse v11.0, was investigated as a possibility for standardized automatic contouring. Smart segmentation is a tool that automatically contours critical structures, organs at risk, and target volumes on unmarked CT images. Smart Segmentation contains a library of expert‐contoured templates which were contoured on actual patient CT images for various disease types and stages.

#### C.3 Treatment plan design

Treatment plan design was also geared toward standardization and reproducibility. Dose constraints for both the plan target volume and organs at risk would have to be based on published standards. RTOG protocols were investigated as possible templates. Field setup (i.e., gantry and collimator angle) was derived from proven clinical templates within our own institution.

#### C.4 Dosimetric tools and analysis

##### C.4.1 Planar dosimetry

Gafchromic EBT3 film was used to collect planar dose profiles. Gafchromic film was selected because it is self‐developing and essentially nonsensitive to short‐term light exposure. Gafchromic film does not require a jacket to prevent exposure by visible light and therefore can be laser‐cut to virtually any shape the user desires, such as the contour of our film planes in phantom. Also, conventional radiographic film requires a processor, something that is being phased out in many oncology centers.

##### C.4.2 OSLD dosimetry:

Landauder nanoDot OSLDs were one of the dosimeters used for point dosimetry. OSLDs are becoming the gold standard for point dosimetry where ion chambers are not feasible (i.e., *in vivo* dosimetry or complex phantom designs). IROC Houston, for example, has transitioned from TLDs to OSLDs for credentialing due to ease of use and greater accuracy. Before OSLDs could be used in a reliable, reproducible manner a series of preliminary measurements had to be acquired. The response of individual dosimeters to a known dose had to be determined vs. the batch average response. A calibration curve was generated by exposing groups of OSLDs to several known doses. Additionally, variation of response with angle of incidence of primary beam relative to plane of detector was investigated.^(8)^

##### C.4.3 CBCT dose

Image guidance is a vital part of clinical workflow in radiation therapy and absolutely needed to be included in this end‐to‐end test. Cone‐beam CT, the most common image guidance modality for IMRT, contributes dose to the overall plan delivery which is not accounted for in the treatment planning system. Response of both film and OSLDs to this dose has to be quantified and subtracted from measurements or an apparent overdose would be observed compared to planned dose.

#### C.5 Analysis

Film dose planes were compared to planning system dose planes using composite gamma index. Gamma index was selected in order to obtain reliable results in both high‐dose gradient and low‐dose gradient regions. While dose difference analysis breaks down in high‐dose gradient regions, distance‐to‐agreement is not meaningful in low‐dose gradient regions.^(9)^ OSLD and ion chamber measurements were compared to point doses from treatment planning system using the dose comparison methodology from AAPM Task Group Report 119^(10)^ in order to utilize a widely accepted comparison technique.

## III. RESULTS

### A. Final phantom design

**Figure 1:**
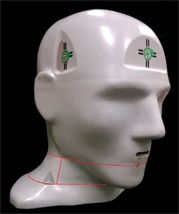
End‐to‐end phantom with film plane locations highlighted in red.

**Figure 2:**
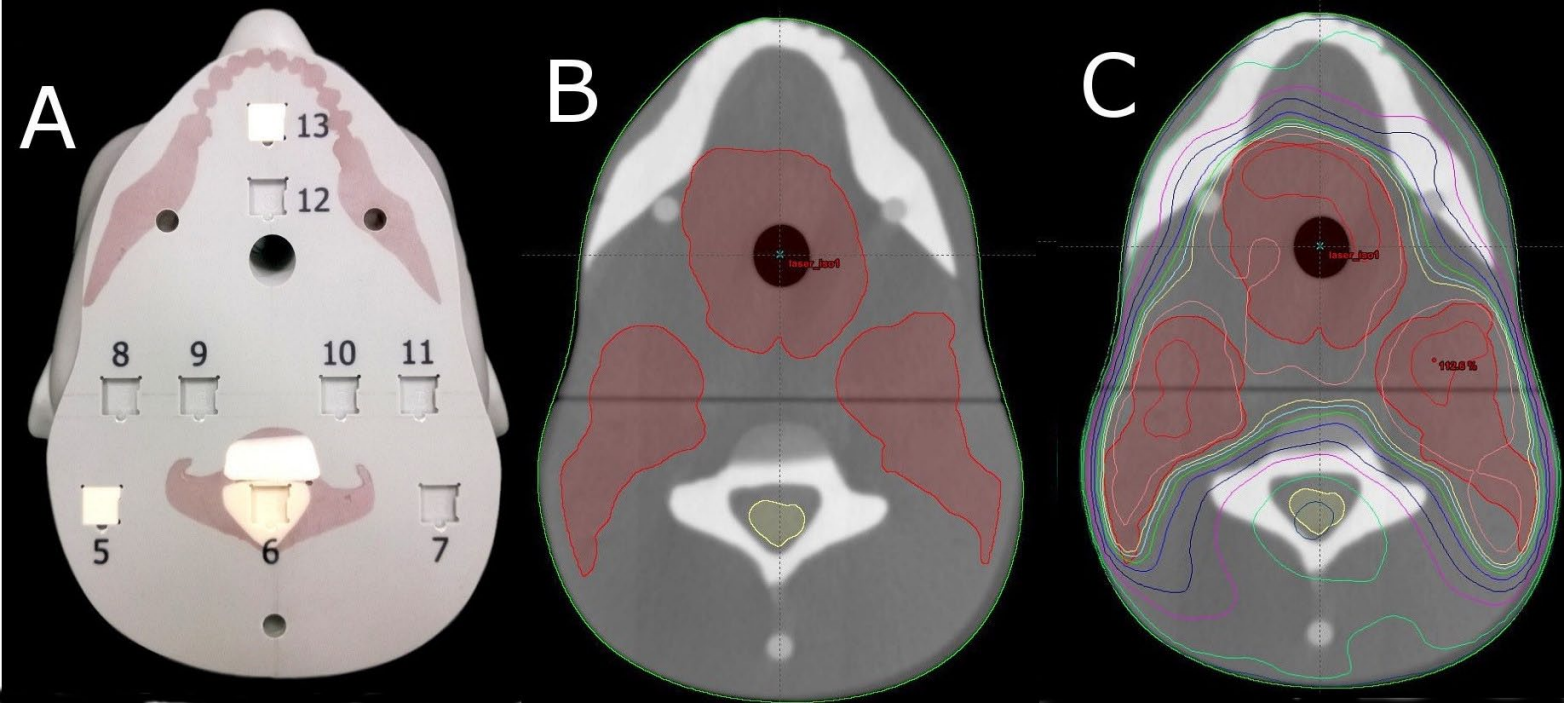
Superior axial film plane: (a) photograph of phantom, observed inferior‐superior. Red structures indicate bone‐equivalent material, numbers correspond to OSLD locations, with location 5 and 13 showing a tissue‐equivalent plug; (b) CT image of same plane in phantom with PTV and cord structure overlay; (c) CT image with isodose distribution.

**Figure 3:**
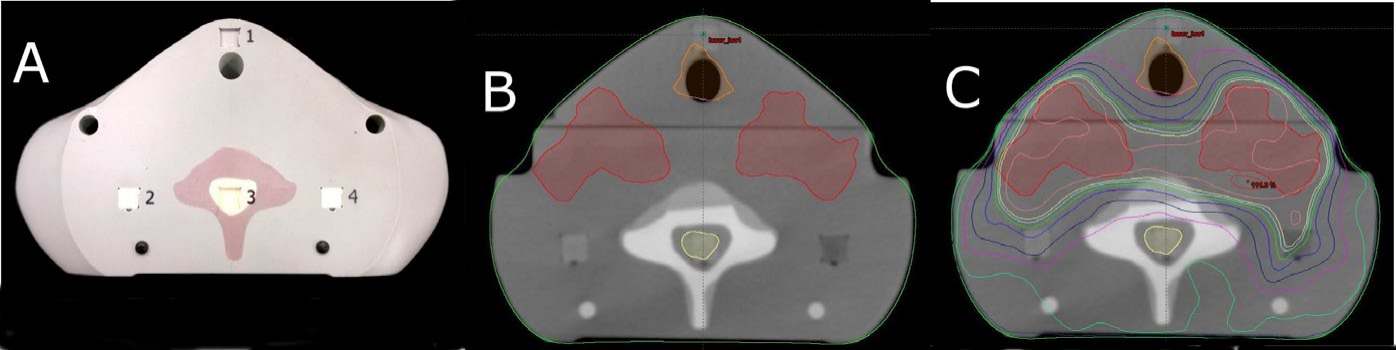
Inferior axial film plane: (a) photograph of phantom, observed superior‐inferior; (b) CT image of same plane in phantom with PTV and cord structure overlay; (c) CT image with isodose distribution.

**Figure 4:**
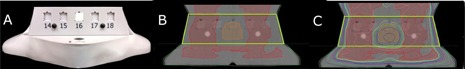
Coronal film plane: (a) photograph of phantom, observed anterior‐posterior; (b) CT image of same plane in phantom with PTV and cord structure overlay;)c) CT image with isodose distribution (film plane highlighted in each CT image).

Figures 1 to 4 display the final phantom design. Final phantom design successfully incorporated all preliminary design characteristics without sacrificing heterogeneities anywhere in the phantom. The design incorporates two axial and one coronal film planes which were created with two axial cuts across the entire phantom and one coronal cut between the two axial planes. This design allows for film dosimetry in three dimensions, including within high‐dose gradient regions (i.e., spinal cord and near parotid glands) while preserving the phantom's “bone” structures. Placement of ionization chamber locations was limited by the film planes; four locations were placed (one in the cord and three in likely PTV locations) below the inferior axial film plane. OSLD locations (a total of 18) were placed adjacent to film planes for point dosimetry. A large number of OSLD locations were included to maximize the versatility of the phantom. When implemented in a clinical end‐to‐end test, OSLD measurements in high‐dose gradient regions, where point measurements are particularly difficult, would not be included in the final analysis.

### B. End‐to‐end design

**Figure 5:**
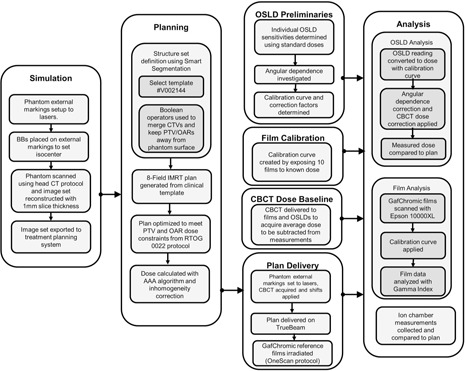
Flowchart for end‐to‐end test.

Figure 5 presents the work flow of the end‐to‐end test with descriptions of the individual components. Details of the specific components are presented below.

#### B.1 Simulation

The end‐to‐end phantom was imaged on a Siemens Sensation CT Simulator. Phantom's external markings were aligned to lasers. Additionally, BBs were placed on intersections of external crosshairs to provide a redundant method for locating laser isocenter in the treatment planning system. Ahead scan protocol was used with 120 kVp and 320 mAs. Images were reconstructed to a 1 mm slice thickness. Image set was imported to Eclipse treatment planning system.

#### B.2 Segmentation and treatment planning

Eclipse Smart Segmentation was used to contour the CTV and organs at risk with template number V002144. This structure set contained two CTVs: one for primary tumor at base of tongue and one for prophylactic bilateral neck irradiation. Both CTV structures were applied to the phantom image set. The following OARs were imported from the Smart Segmentation template: brain stem, larynx, left and right parotid glands, and spinal cord. PTVs were created by adding a 3 mm margin to the CTVs. In order to limit PTV and OAR contours to 2 mm from surface and deeper, a body margin structure was created by applying a 5 mm internal margin to the body contour. Boolean operators were used to create new contours for PTVs and parotid glands which excluded any contour outside of the “body margin” structure. Finally, the modified PTVs were merged into one combined PTV with Boolean operators.

An eight‐field static gantry IMRT plan was created from a clinical head and neck template which used 7° collimator angle for all beams and the following gantry angles: 35°, 80°, 125°, 160°, 200°, 245°, 275°, and 315°. Couch was rotated to 7° and 353° for the 80° and 275° fields,

respectively. Plan was designed to meet RTOG protocol 0022. Prescription dose of 66 Gy in 30 fractions was selected. Plan was optimized to meet all PTV and OAR dose constraints as outlined by RTOG protocol. After optimization, dose was calculated using the analytic anisotropic algorithm v11.0 with inhomogeneity correction. Evaluation of the completed plan showed that all PTV and normal tissue dose constraints were met.

#### B.3 Setup and delivery

The plan was delivered on a Varian TrueBeam equipped with a high‐definition MLC. Phantom was set up by aligning external markings to lasers. Couch was shifted by the difference between plan isocenter and intersection of external markings. Cone‐beam CT was acquired using the head scan protocol (full fan, half sweep, 100 kVp and 150 mAs) and automatching was used to determine shifts. Shortly after plan delivery, two film patches were irradiated to known doses in solid water. This was done for linear scaling of phantom films, which will be discussed in the film analysis section.

#### B.4 Planar dosimetry

Gafchromic EBT3 film was used to collect planar dose profiles. All films were laser‐cut by the phantom manufacturer to fit specific film locations in phantom. Film data were digitized with an Epson 10000XL flatbed scanner and analyzed with FilmQA Pro (Ashland Advanced Materials). All films were scanned into a. TIF format in transmission mode with scanner set to 72 dpi resolution and 48‐bit tricolor (RGB) mode with all color corrections turned off.

A calibration curve was generated by irradiating 10 film patches to 10 known doses in solid water phantom at $d_{\text{max}}$ with a 10×10 cm field. Films were scanned as one group and imported into FilmQA Pro as a Film Calibration object. Dose values were entered and a dose calibration curve was generated using the linear reciprocal function

(1) X(D)=a+b/(D−c)

where *X(D)* is response as function of dose, and *a, b*, and *c* are constants.

Each phantom film was scanned with the two reference films which were irradiated after plan delivery as recommended in the OneScan protocol developed by Lewis et al.^(11)^ OneScan protocol helps to eliminate scanner variability and environmental factors affecting the film results. Film bitmaps were automatically converted to dose maps using the linear reciprocal calibration with triple‐channel uniformity optimization. ROIs were added to reference film patches, region type changed to “calibration region”, and actual delivered dose manually entered for both calibration regions. Linear scaling was turned on in dose mapping method menu and dose map was rebuilt. Fiducial markers were added to each dose map for image registration; fiducial markers were placed on regions in dose map which corresponded to physical registration markers on the film.

To account for CBCT dose, films were irradiated with only the setup CBCT field and converted to dose maps using the same technique as used for the full end‐to‐end test. It should be noted that this technique does not account for the actual dose to phantom from the CBCT, due to the energy dependence of EBT3 film. Our technique instead accounts for the apparent additional dose seen in a film analyzed with a megavoltage calibration; which allows for the subtraction of this apparent overdose, leaving only the therapeutic dose information. The image summation tool was used to subtract the CBCT dose maps from each end‐to‐end dose map, which effectively subtracted the CBCT component of dose, leaving only the dose that the film was exposed to with the therapeutic fields. Apparent CBCT contribution to film dose was determined to average approximately 2 cGy with four CBCT exposures to the same film, or 0.5 cGy for an individual CBCT exposure.

Plan dose was exported from Eclipse as a single DICOM file containing the entire 3D volume dose and imported into FilmQA Pro. A dose plane was selected for each film location by selecting the appropriate depth based on coordinates of the isocenter and coordinates of the film plane. A resolution of 72 dpi was selected for the dose plane to be extracted. Fiducial markers were placed in the DICOM dose map based on TPS coordinates of registration markers used with the film. DICOM dose map and film dose map were registered by fitting the patterns of fiducial markers in the two dose maps. Using this registration technique, submilimeter alignment between film and DICOM dose maps can be expected. Rectangular ROIs were selected inside dose maps (to avoid regions where DICOM data exist but film data do not). Measured dose maps were compared to planned dose using gamma index normalized to global maximum with 3%/3 mm and 3%/2 mm, and 3%/1 mm passing criteria with 10% maximum dose threshold.

#### B.5 Absolute dosimetry

##### B.5.1 OSLD dosimetry

Initially all OSLDs were individually irradiated to 1 Gy with a 6 MV beam in solid water at a depth of 1.5 cm in a 10×10 cm field. OSLD response was read with an InLight microStar Reader (Landauer Inc.) with four readings per each individual OSLD measurement in order to reduce statistical uncertainty of the microStar system. Individual correction factors (ICFs) were calculated for each OSLD by dividing individual response by the average response of the batch.^(12)^ ICF measurements were repeated in order to determine variation between measurements (three separate irradiation/measurement cycles in total). OSLDs were reset after each irradiation/measurement by light annealing in bright light conditions for 24 hrs to completely clear all electron traps. A calibration curve was created by irradiating groups of OSLDs to five known dose points (five dosimeters per dose point), again in solid water at 1.5 cm depth and in a 10×10 cm field. Adjusted signal was calculated by dividing signal from each dosimeter by its individual sensitivity factor. Dose was plotted vs. adjusted signal, a linear fit and a 2nd order polynomial fit were applied and compared. Polynomial fit was found to be the better option based on plot of residuals and the intercept being much closer to zero cGy at zero counts.

Initial phantom measurements were taken with OSLDs present in all 18 locations over three trials to determine baseline results. Several locations which gave consistent results, indicated by low standard deviations, were selected for additional analysis. Several phantom measurements (five total) were taken in these locations only, one immediately after the other. This was done in order to eliminate day‐to‐day variations in linac performance, effectively leaving only statistical variation among individual OSLDs, statistical fluctuation in reading OSLDs, setup uncertainties, and the actual difference between plan dose and delivered dose. Phantom measurements were converted to “adjusted counts” by dividing raw counts by individual dosimeter sensitivity. Calibration formula was applied to convert adjusted counts to measured dose. Percent difference was calculated using TG‐119 formalism^(10)^ [(measured dose−planned dose)/prescribed dose]×100.

To account for CBCT dose, several measurements were taken in all OSLD locations while delivering only four CBCT setup fields. The apparent CBCT dose to the OSLDs was calculated by applying the same calibration formula and sensitivity factors used in the calculation of MV dose. Apparent CBCT dose to OSLDs ranged between 3.9 and 4.4 cGy over a total of four CBCT exposures, for a correction between 0.98 and 1.1 cGy for individual OSLD locations. Average apparent CBCT dose for each point was subtracted from each end‐to‐end delivery in order to account for dose from CBCT.

Another factor which was investigated was the possibility of decreasing OSLD response with angle of primary beam. OSLDs were positioned with plane of dosimeter disc perpendicular to primary beam for known‐dose irradiations to build calibration curve. In phantom most OSLD locations have the plane of OSLD parallel to incoming primary beam. A phantom was constructed which allowed us to vary the angle of OSLD vs. an open field without changing the depth of effective point of measurement. OSLDs were irradiated in 10×10 cm field at angles of 0° (beam perpendicular to disc), 30°, 45°, 60°, and 90°, with five dosimeters per location. Response was normalized to average response for 0° measurements and plotted vs. angle.

**Figure 6:**
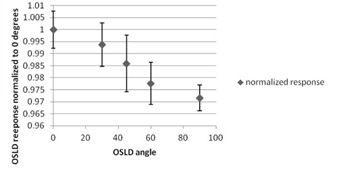
Plot of normalized OSLD response vs. angle of incidence of the primary beam.

Angular dependence of OSLD response was found to be up to −3% at 90° (dosimeter parallel to primary beam). For locations 1 to 13, which are always at 90° to primary beam, a correction factor of 1.03 was applied. Locations in the coronal plane had mean beam angle of 50°, based on individual beam angles. A correction factor of 1.015 was applied to these locations. Figure 6 shows relative OSLD response at different angles of incident primary radiation.

##### B.5.2 Ionization chamber dosimetry

An A1SL ionization chamber was calibrated each day before phantom measurements by irradiating to 1 Gy in solid water at 1.5 cm depth in a 10×10 cm field. The charge collected was used to create a charge‐to‐dose calibration factor for phantom measurements. Phantom measurements were collected with one chamber with subsequent back‐to‐back plan deliveries to measure dose in all four locations. Measured dose was compared to plan dose using TG‐119 formalism [(measured dose−planned dose)/prescribed dose]×100.

### C. Preliminary measurements and results of the end‐to‐end procedure

Preliminary results show that film plane locations provide desired coverage within a typical clinical PTV as well as OARs such as the spinal cord and larynx. Film plane locations also included high‐dose gradient regions, which provide information about geometric accuracy. OSLD locations were sufficient to provide a significant number of point‐dose measurements despite a few OSLDs falling in high‐dose gradient regions. While ion chamber locations were limited by technical details, locations that could be incorporated still provide a reasonable second check to verify reliability of OSLD measurements. Multiple OSLD locations and ion chamber locations yielded highly reproducible point‐dose measurements.

#### C.1 Baseline measurement results

##### C.1.1 Ionization chamber measurements

Ion chamber dose measurements were within 3% of the expected values from the treatment planning system. The largest deviation was −2.76%, which was located at midline in between the PTV contours, anterior to the spinal cord and posterior to the trachea. Data for individual ion chamber locations are presented in Table 1.

**Table 1:** Ionization chamber data averaged over five total measurements

*Ion Chamber Location*	*Average Measured Dose (cGy)*	*Percent Difference From Plan Dose*	*SD*
Spinal cord	117.4	0.16	0.32
Anterior of cord	234.0	−2.76	0.27
Right of cord	244.5	1.42	0.19
Left of cord	239.4	0.27	0.16

##### C.1.2 Film results

Gamma analysis of all films (four in total for each location) yielded >99% gamma pass rates at 3%/3 mm passing criteria, ~96.2% at 3%/2 mm, and 88.3% at 3%/1 mm. Complete film results are presented in Table 2.

**Table 2:** Results of film gamma analysis averaged over four films for each location

*Location*	*Passing Criteria*	*Passing Rate (%)*	*SD (%)*
Superior axial plane	3%/3 mm	99.8	0.23
	3%/2 mm	92.9	2.4
	3%/1 mm	83.2	3.8
Inferior axial plane	3%/3 mm	98.8	1.3
	3%/2 mm	97.9	1.2
	3%/1 mm	91.1	4.3
Coronal plane	3%/3 mm	99.6	0.03
	3%/2 mm	97.7	1.1
	3%/1 mm	90.7	2.04

##### C.1.3 OSLD results

**Figure 7:**
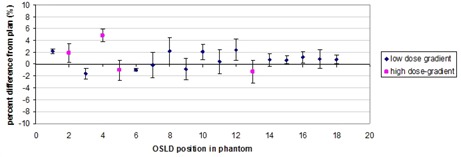
Plot of OSLD percent difference from plan dose for each OSLD location with error bars indicating SD of readings. Pink square symbols represent locations which were suspected to be high‐dose‐gradient regions.

For an individual measurement of an OSLD, the standard deviation (SD) among four readings was determined to average 1.07% across the batch. Average SD among three sensitivity measurements, with each measurement averaged over four instrument readings, was calculated to be 1.31% across the batch. Average difference from prescription dose for all locations (averaged over four measurements with four readings for each measurement) was 0.80 % with a SD of 1.33%. Excluding suspected high‐dose gradient points average difference was 0.68% with average SD of 1.29%. For multiple same‐day in‐phantom measurements average difference from Rx dose was 0.41%, with average SD of 1.26%. Standard deviations were reported as the deviation of the percent difference from plan dose among all measurements for one OSLD location. Standard deviation among phantom measurements was found to be within uncertainty of OSLDs and microStar system, indicating that the main source of error was not from the plan delivery itself. Figure 7 shows the percent difference between measured and planned doses at all OSLD locations. Table 3 shows OSLD results from repeated same‐day measurements at selected OSLD locations.

**Table 3:** OSLD results for repeated same‐day measurements for selected points. Measurements reported are for six OSLD locations with five total measurements in each location. A single measurement for one OSLD is the average of four readings of that OSLD in a microStar reader

*OSLD Location*	*Percent Difference From Plan*					*Average*	*SD*
1	0.12	−0.60	0.84	2.15	2.74	1.05	1.39
9	−1.20	−2.92	−0.30	0.89	−0.19	−0.74	1.42
11	0.18	2.17	0.43	−0.59	0.01	0.44	1.04
12	2.39	4.24	−0.59	0.07	1.59	1.54	1.92
16	−1.23	1.58	−1.19	0.05	0.13	−0.13	1.16
18	0.01	−0.43	0.22	1.36	0.26	0.28	0.66

## IV. DISCUSSION

Our results indicate that our end‐to‐end (E2E) test can provide reproducible geometric and dosimetric information about the accuracy of the TrueBeam system in a clinically realistic head and neck case. Our phantom design allows for excellent coverage of a typical H&N treatment volume and OARs in terms of both point dosimeters and planar film dosimetry without sacrificing realistic bony anatomy and air cavities. This realistic phantom design allows our E2E test to account for the accuracy of treatment planning algorithms in regard to heterogeneity corrections, which is an improvement over tests which utilize homogeneous phantoms. The reproducibility of our results means that our end‐to‐end procedure can be potentially implemented as a standardized, routine part of a comprehensive quality assurance program. Pass/fail criteria will have to be determined before this implementation can be made, however. Testing by personnel not involved in initial test design will be required to help determine these criteria.

While a successful design overall, final phantom design was slightly limited due to technical limitations of the production process. Ion chamber locations were limited to below the inferior axial film plane due to several factors. Penetrating the film planes would necessitate additional cuts to the custom films and placing ion chambers across a plane cut across the entire phantom would essentially amount to placing a sensitive ion chamber along a potential stress point. One potential solution would have been to design some form of inserts, similar to the ball‐cube design, instead of film planes across the entire phantom. However, such a design would prove difficult to manufacture while preserving the “bone” and “sinus” anatomical structures of the phantom. We determined that preserving these structures as well as placing film locations across the entire axial profile was more important than more versatile ion chamber locations. Focusing on OSLD placement for point dosimetry also gave the advantage of being able to simultaneously collect data in many locations.

When compared with the only standardized E2E test for H&N treatment, the IROC Houston credentialing test, our procedure and phantom has several potential advantages. Our phantom incorporates realistic bony anatomy whereas the IROC Houston phantom is water‐filled outside of the test cube. The realistic bone structures can potentially contribute to improved testing/ analysis of the heterogeneity corrections of the treatment planning system. Also our test relies on more complex, more realistic target volume and organ‐at‐risk structure, potentially pushing the planning system to its limits just as a complex clinical plan would. The most significant improvement over the IROC Houston H&N test is that our E2E test is meant to be an “in‐house” quality assurance test, which can be incorporated into routine QA procedure which can identify potential problems on a continuous basis, instead of being something that is completed only once to pass credentialing. A potential disadvantage, when compared to the IROC Houston test, is that our E2E test relies on more complicated structure contouring based on Smart Segmentation (and therefore the user's ability to use this technique) and the ability of the individual user to correctly analyze their results. However, with thorough step‐by‐step documentation and a wide range of published baseline results, these factors can be minimized.

Having determined the baseline accuracy of our E2E test our next immediate step is to baseline results of the end‐to‐end procedure across multiple sites using TrueBeam linear accelerators. This will allow us to determine (i) reproducibility of end‐to‐end procedure across multiple sites; (ii) difficulties that an outside user may have with our instructions and fine‐tune instructions; and (iii) common modes of failure and how to reduce them. A potential future development from this project would be to perform a comparison of our E2E test against current IMRT quality assurance/commissioning methods with regard to effectiveness of finding deliberately introduced errors in order to gauge sensitivity of finding actual error. Another area of potential future work is to apply our methodology to designing similar end‐to‐end procedures for other locations (i.e., lung, prostate).

## V. CONCLUSIONS

Our phantom design was successful in obtaining sufficient point and planar dosimetric information while preserving anatomical heterogeneities. Our baseline results demonstrate that our testing methodology can yield highly accurate, highly reproducible results.

## ACKNOWLEDGMENTS

The authors would like to thank Varian Medical Systems for providing support and funding for this project.

## COPYRIGHT

This work is licensed under a Creative Commons Attribution 4.0 International License.
